# Cardiac Autonomic Modulation during on-Call Duty under Working Hours Restriction

**DOI:** 10.3390/ijerph17031118

**Published:** 2020-02-10

**Authors:** Jien-Wen Chien, Chung-Yen Chen, Sheng-Hsuan Lin, Shih-Wen Lin, Yu-Hsuan Lin

**Affiliations:** 1 Department of Pediatrics, Changhua Christian Hospital, Changhua 50006, Taiwan; 103207@cch.org.tw; 2 Institute of Environmental and Occupational Health Sciences, College of Public Health, National Taiwan University, Taipei 10055, Taiwan; 3 Department of Environmental and Occupational Medicine, National Taiwan University Hospital, Taipei 10048, Taiwan; ccyares@gmail.com; 4 Institute of Statistics, National Chiao-Tung University, Hsinchu 30010, Taiwan; shenglin@stat.nctu.edu.tw (S.-H.L.); a258bc106@gmail.com (S.-W.L.); 5 Institute of Population Health Sciences, National Health Research Institutes, Miaoli 35053, Taiwan; 6 Department of Psychiatry, National Taiwan University Hospital, Taipei 10048, Taiwan; 7 Department of Psychiatry, College of Medicine, National Taiwan University, Taipei 10048, Taiwan; 8 Institute of Health Behaviors and Community Sciences, College of Public Health, National Taiwan University, Taipei 10055, Taiwan

**Keywords:** autonomic nervous system, Heart rate variability, medical resident, on-call duty, working hours restriction

## Abstract

**Background:** Medical residency is a time of high stress and long working hours, which increase the risk of cardiovascular disease. This study aimed to investigate the autonomic modulation of resident physicians throughout the on-call duty cycle. **Methods:** Spectral analysis of heart rate variability (HRV) was used to compute cardiac parasympathetic modulation (high-frequency power, HF) and cardiac sympathetic modulation (normalized low-frequency power, LF%, and the ratio of LF and HF, LF/HF) of 18 residents for a consecutive 4-day cycle. **Results:** Male residents show reduced cardiac sympathetic modulation (i.e., higher LF/HF and LF%) than the female interns. Medical residents’ cardiac parasympathetic modulation (i.e., HF) significantly increased on the first and the second post-call day compared with the pre-call day. In contrast, LF% was significantly decreased on the first and the second post-call day compared with the pre-call day. Similarly, LF/HF was significantly decreased on the second post-call day compared with the pre-call day. LF/HF significantly decreased on the first post-call day and on the second post-call day from on-call duty. **Conclusion:** The guideline that limits workweeks to 80 h and shifts to 28 h resulted in reduced sympathetic modulation and increased parasympathetic modulation during the two days following on-call duty.

## 1. Introduction

In the traditional extended-duty shift model, each new admission to the on-call shift experiences a reduction in the amount of on-call sleep and an increase in the total shift duration [1]. Recent prospective studies and meta-analyses also suggest that long working hours [2] and shift work [3] increase the risk of cardiovascular disease (CVD). In addition, on-call duty among medical residents is characterized by sleep deprivation and stressful working conditions. Sleep deprivation alters cardiovascular reactivity to acute stressors [4] and increases the incidence of CVD [5,6,7]. Systematic reviews have reported that such long working shifts and erratic schedules lead to acute and chronic sleep deprivation in resident physicians, resulting in numerous adverse consequences in patient care [8].

In 2011, the Accreditation Council for Graduate Medical Education (ACGME) revised medical resident duty-hour policies, limiting resident workweeks to 80 h and shifts to 28 h. These policies were based on studies of the effects of sleep deprivation on human performance and specifically on the effect of extended shifts on resident performance [9]. In Taiwan, similar policies by the Ministry of Health and Welfare limited workweeks to 88 h; and in 2013 there were three versions of limits on consecutive work hours: 24, 28, and 32 h [10]. Several evaluations of these limitations on working hours found essentially no differences in patient outcomes after their implementation [11,12,13,14,15]; however, few studies have investigated the impact of the current working hours policies on physicians’ physiologies, especially cardiovascular reactivity [16,17,18].

A physiological measurement of cardiac function, which varies with stress, is heart rate variability (HRV). The autonomic nervous system is the primary regulator of heart rate. Rhythmic fluctuations in the frequency of impulse conduction along the vagus nerves, modulated by the rate and depth of breathing, result in substantial variations in R-R intervals, a condition known as respiratory sinus arrhythmia [19]. R-R intervals are also affected by mental or physical activity, which reduce the average frequency of the impulses conducted along the vagus nerves and increase the activity of the sympathetic nervous system. The application of HRV analysis has recently gained popularity as a means of quantifying autonomic nervous system (ANS) functioning noninvasively [20,21,22]. Spectral analysis of HRV by Fourier transformation allows categorization into high frequency (HF), low frequency (LF), and very low frequency (VLF) powers. HF is considered to represent vagal control of the heart rate. Because both sympathetic and parasympathetic activities contribute to LF, the ratio LF/HF and normalized LF (LF%) are considered by some investigators to reflect sympathetic modulation [20,21,22].

Our earlier studies have described the long-term [17] and short-term [18] cardiac autonomic modulation among medical interns in workweeks of up to 86.7 h and shifts of up to 33.5 h. Male medical interns with this extremely heavy workload showed a reduced cardiac sympathetic modulation (LF/HF) during duty night work and increased sleep vagal activity (HF) during the first sleep after on-call duty, which corresponded to the Stanford Sleepiness Scales. In a one-year follow-up study [17], interns presented a reduced HRV at 6, 9, and 12 months and cardiac parasympathetic modulation (HF) at 9 and 12 months into their internship, both of which suggested a long-term risk profile of CVD. This study is designed to test the hypothesis that the current duty hours, which limit workweeks to 80 h and shifts to 28 h, have not substantially altered the short-term effects of cardiac autonomic modulation after medical residents’ on-call duty. In addition, most previous studies compared physicians’ HRV indices only during on-call duty and one non-call day rather than a longer post-shift period. The present study had three specific aims: first, to follow, as primary outcome, changes in autonomic functioning of residents through on-call duties, the first post-call day, and the second post-call day by analyzing HF, LF%, and LF/HF, via frequency domain analysis of the HRV; second, to identify these autonomic functional changes during the duty night and post-call rest by controlling the effects of circadian rhythm; and finally, to determine whether any gender differences occur with respect to the physiological impact of on-call duty.

## 2. Materials and Methods

### 2.1. Participants

A total of 18 pediatric residents trained at a hospital in middle Taiwan were voluntarily recruited. Of these, 8 males and 4 females were in the postgraduate year (PGY) program, and the other 3 males and 3 females were pediatric residents. All participants were healthy adults, aged 28.1 ± 2.5 (mean ± SD) years. They did not have hypnotic drug abuse or excess alcohol, caffeine, or nicotine consumption during the entire period of study. None of them had any medical condition known to involve sleep or the ANS, such as a psychiatric or cardiovascular disease.

**Figure 1:**
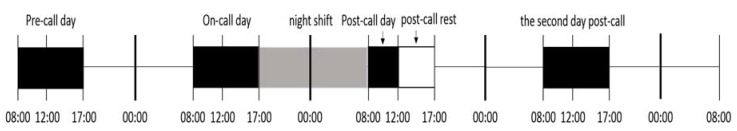
Participants underwent electrocardiographic (ECG) recording for a four-day cycle: the pre-call day, the on-call day, and two post-call days. Each on-call day comprised of routine work from 08:00 to 16:59 (9 regular working hours daily) followed by on-call duty from 17:00 to 07:59 (15 h). Per the “post-call rest” policy of the hospital, the residents continued to work from 08:00 to 11:59 (4 h), a total of 28 consecutive hours during the on-call cycle. Circadian period was separated into three groups: morning (08:00–11:59), afternoon (12:00–16:59), and night (17:00–23:59). The pre-call day and the morning period were used as self-control references.

During their pediatric residency training, the participants worked approximately 70.6 to 76.8 h a week, including 28 consecutive work hours and an average of 6 to 9 on-call duties monthly. Each on-call day comprised routine work from 08:00 to 16:59 (9 regular working hours daily), followed by on-call duty from 17:00 to 07:59 (15 h). Per the “post-call post meridiem off” policy of the hospital, the residents continued to work from 08:00 to 11:59 (4 h), and the 28 consecutive hours during on-call cycle would meet the working hours limitation made by the Ministry of Health and Welfare (Figure 1). Generally, 7–9 on-call days per month were scheduled for the PGY residents and 6 on-call days for the pediatric residents.

Participants were given a detailed description of the study; and individual informed consent was obtained in written form. They were aware of this study’s intention to obtain assessments of their autonomic functioning and to monitor their activity and rest cycles. The study was conducted between December 2015 and June 2016. The study protocol was approved by the Ethics Committee of this hospital.

### 2.2. Heart Rate Variability

Participants underwent electrocardiographic (ECG) recording by means of a miniature physiological signal recorder (TD1, Taiwan Telemedicine Device Company, Taipei, Taiwan) [23]. The small size (5.2 × 3.1 × 1.2 cm) and low weight (11 g) of the recorder produced minimal interference with work or stress on the participants. Each resident was monitored by the two-lead digital ambulatory ECG recorder with an accelerometer, which was attached at 23:59 on the day before the shift (pre-call). The device continuously recorded throughout the on-call day and the following two days (the post-call day and the second post-call day) until removal at the end of the second post-call day.

The power spectral density was calculated by computerized fast Fourier transformation to evaluate the R-R tachograms by frequency-domain measures. The R-R signals to be analyzed were truncated into successive 64 s (4096 points) time segments (windows or epochs) with 50% overlapping. A Hamming window was applied to each time segment to attenuate the leakage effect [24]. We also handled the artifacts by the R-R rejection procedure. A temporary mean and SD of all R-Rs were first calculated for standard reference. Each R-R was then validated. If the standard score, i.e., (value-mean)/SD, of an R-R value exceeded three, it was considered erroneous or nonstationary and was rejected [25]. The average percentile of R-R rejection according to this procedure was 1%. Our algorithm then estimated the power density of the spectral components based on fast Fourier transformation every 5 min. The resulting power spectrum was corrected for attenuation resulting from sampling and the application of the Hamming window. The power spectrum was subsequently quantified into the standard frequency-domain measurements as reported previously, including the variance of the R-R interval values, VLF power (0.003–0.04 Hz), LF (0.04–0.15 Hz), HF (0.15–0.40 Hz), total power (TP), LF/HF, and normalized LF (LF%). LF% was calculated from LF/(HF + LF) × 100. The variance of the R-R interval, LF, HF, and LF/HF values were logarithmically transformed to correct for their skewed distributions [25].

### 2.3. Sleep Data and Accelerometry

We obtained sleep data using a weekly sleep log together with an accelerometer that was attached to the recorder. Acceleration values were stored in the flash memory for each axis, namely X (mediolateral), Y (vertical), and Z (anteroposterior). Each axis had a sampling frequency of 125 Hz and could detect accelerations ranging from −3G to 3G. A vectorial magnitude was calculated as $\sqrt{x^{2}+y^{2}+z^{2}}$. The quantified magnitude of physical activity was estimated by calculating the root mean square of the vectorial magnitude for each time period (epoch). We applied the previously described zero-crossing method to count the number of times per epoch of sleep time that the activity signal level approached zero, 0.004087 g [18]. In our study, the ECGs recorded during nocturnal sleep were excluded.

### 2.4. Statistical Analysis

Heart rate continuously changes within a person and was repeatedly measured by heart rate monitoring. Due to the auto-correlations for all measurements within the same participant, it is unreasonable to treat such measurements as fully independent among participants. Linear mixed effect models were used for the statistical analysis, in which all major determinants—such as gender, duty day, and circadian period—were included as fixed effects, while the baseline differences of heart rate among all individuals were seen as a random effect. Four working days were named as the pre-call day (Day 0), the on-call day (Day 1), the first post-call day (Day 2), and the second post-call day (Day 3). The circadian period was separated into three groups: morning (Time 0, 08:00–11:59), afternoon (Time 1, 12:00–16:59), and night (Time 2, 17:00–23:59). Four measurements of outcome of interest are R-R interval, HF, LF%, and the ratio of LF to HF. We build a mixed regression model as follows:

(1) $$HRV=b_{0i}+\beta_{1}\;Male_{ij}+\beta_{2}\;Day1_{ij}+\beta_{3}\;Day2_{ij}+\beta_{4}\;Day3_{ij}+\beta_{5}\;Time1_{ij}+\beta_{6}\;Time2_{ij}+\varepsilon_{ij}$$

where

(2) $$b_{0i}\;\sim \;N\left(0,\;\sigma_{b}{}^{2}\right),\varepsilon_{ij}\;\sim \;N\left(0,\;\sigma_{\varepsilon }{}^{2}\right).$$

For model 1, $b_{0}$ is a random intercept; $\beta_{1}$ is the effect of male versus female; $\beta_{2}$ is the effect of the on-call day versus that of the pre-call day; $\beta_{3}$ is the effect of the post-call day versus that of the pre-call day; $\beta_{4}$ is the effect of the second post-call day versus that of the pre-call day; $\beta_{5}$ is the effect of the afternoon (12:00–16:59) versus that of the morning (08:00–11:59); $\beta_{6}$ is the effect of the night (17:00–23:59) versus that of the morning (08:00–11:59).

Because participants were exhausted during and after duty, the HRV measurements then were expected to be abnormal compared with other intervals after adjusting the duty day and circadian period. Therefore, we built another model to specifically quantify the effect of duty night (Day1, time2) and post-call rest (Day2, time1) as follows:

(3) $$HRV=b_{0i}+\beta_{1}\;Male_{ij}+\beta_{2}\;Day1_{ij}+\beta_{3}\;Day2_{ij}+\beta_{4}\;Day3_{ij}+\beta_{5}\;Time1_{ij}+\beta_{6}\;Time2_{ij}+\beta_{7}\;duty_{night}{}_{ij}+\beta_{8}\;post_{call_{rest}}{}_{ij}+\varepsilon_{ij},\;$$

where

(4) $$\;b_{0i}\;\sim \;N\left(0,\;\sigma_{b}{}^{2}\right),\;\varepsilon_{ij}\;\sim \;N\left(0,\;\sigma_{\varepsilon }{}^{2}\right).\;$$

For model 2, the interpretations of $b_{0}$, $\beta_{1}$, $\beta_{2}$, $\beta_{3}$, $\beta_{4}$, $\beta_{5}$, $\beta_{6}$ are identical to model 1; $\beta_{7}$ is the effect of the night shift; and $\beta_{8}$ is the effect of post-call rest after adjusting all other determinants. The function glmer in R 3.3.0 was used to implement the mixed model. A *p*-value < 0.05 was considered statistically significant.

## 3. Results

**Figure 2:**
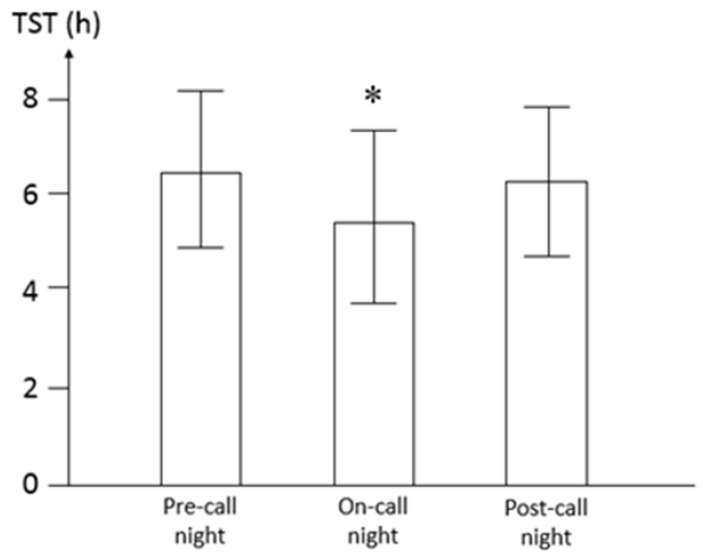
Total sleep time (TST) during the on-call duty cycle. The results are expressed as Mean ± SD. * Residents slept significantly shorter (*p* = 0.015) during on-call duty compared to the pre-call night and post-call night by one-way analysis of variance (ANOVA) test.

Table 1 and Table 2 show that the male residents had higher LF/HF and LF% than the female interns (*p* < 0.001). There is a significantly higher R-R interval, i.e., lower heart rate, during night-time, but no significant difference of HRV indices in spectral analysis, including HF, LF%, and LF/HF. Table 1 shows that the HF significantly increased on the first post-call day (*p* = 0.041) and the second post-call day (*p* < 0.001) compared with the pre-call day. In contrast, LF% significantly decreased on the first post-call day (*p* = 0.001) and the second post-call day (*p* = 0.016) from the pre-call day. Similarly, LF/HF significantly decreased only on the second post-call day (*p* = 0.023) compared with the pre-call day. Table 2 shows that there is no significant difference in any HRV measurements during the night shift (17:00 to 23:59) and post-call rest (12:00 to 16:59) by controlling the effects of gender, duty day, and circadian period. Figure 2 shows pediatric residents slept significantly shorter (*p* = 0.015) during on-call duties (5.44 ± 1.20 h) compared to the pre-call nights (6.50 ± 1.10 h) and post-call nights (6.27 ± 1.02 h).

## 4. Discussion

This is one of the first studies targeting medical residents to evaluate the night shift effects on HRV of long working hours for four consecutive days, including the pre-call day, on-call duty, the first post-call day, and the second post-call day. This is also the first study to investigate the physiological impacts of on-call-duty since the implementation in Taiwan of a new policy on duty hours for medical residents. In this study, decreased sympathetic activities (LF/HF and LF%) and increased vagal activities (HF) indicated the exhaustion stage of stress adaptation, but the dynamic patterns differed. Our findings lend evidence to support our previous study, which found similar patterns of HRV after on-call duty in medical interns [18], though the increased HF and decreased LF/HF occurred at different times. On-call duty among physicians is characterized not only by long working hours but also by sleep deprivation. Therefore, we demonstrated the impact of on-call duty on HRV by controlling for the effects of circadian rhythm. In addition, the present study, as well as our previous studies [16,17,18], use the within-subjects design to eliminate any inter-individual differences. Considering the complex interactions among heart rate, respiration, and blood pressure regulation during social–emotional tasks, this within-subjects design was recommended as the most appropriate method of HRV in bio-behavioral research [26]. Specifically, our previous study showed that medical interns presented lower LF/HF during night-time shifts than during daytime work [18]. This lower LF/HF might result from long consecutive working hours or circadian rhythm [26]. In the present study, we demonstrated significantly reduced LF/HF and LF% after on-call duty by controlling for circadian rhythm.

The dynamics of LF/HF, which may represent cardiac sympathetic modulation, corresponded to the three stages in the general adaptation syndrome model of stress: alarm, resistance, and exhaustion [27]. During on-call duty, sympathetic activities (LF/HF) slightly increased from pre-call days, that is, the alarm stage. LF/HF decreased significantly during the first post-call day from on-call duty; but the level of LF/HF did not differ from that during the pre-call day. These findings were consistent with the resistance stage in the general adaptation syndrome model of stress. The significant reduction in LF/HF not only indicated the exhaustion stage but also implied that the current duty-hour restriction did not provide enough rest to allow medical residents to recover from this stress. The on-call duty reduced LF/HF both in the present study and our earlier study [18]. Medical residents’ LF/HF and LF% decreased on the two post-call days in this study, but medical interns’ LF/HF decreased during the working night during on-call duty in our earlier study [18]. Interns, as freshmen trainees within hospitals, typically work the greatest number of hours per week. Therefore, it is conceivable that interns’ LF/HH reduced during duty night work, which was earlier than residents’ post-duty LF/HF reduced in the present study. It is noteworthy that LF/HF is an indicator of ventricular tachycardia (VT) and sudden cardiac death in ambulatory 24-h HRV recordings. LF/HF decreased before VT [28] and immediately increased before VT onset [29,30,31].

There are several implications of the slight differences of the post-call reduction in sympathetic indicators, that is, LF% and LF/HF. Firstly, the HF is generally representative of cardiovagal activities, whereas the long-standing controversies of the LF%, LF/HF, and sympathetic indicators are ongoing debates [22,32]. Because the LF component of HRV reflects a mix of sympathetic, parasympathetic activities, and other unidentified factors in this frequency range, there is an inherent methodological limitation to use LF% or LF/HF representing sympathetic activities. Secondly, LF% are more likely to fit parametric assumptions, but LF/HF may show significant skew and kurtosis. Consequently, LF/HF may exhibit an obvious decrease in precision [32,33]. Thirdly, the decreased LF% was significant and LF/HF not during the first post-call day from pre-call day, this might be interpreted as a reduction in relative sympathetic activity. Lastly, we included both LF% and LF/HF of the 5-min recording to measure short-term HRV in this study. In order to successfully approximate the underlying information in bio-signal analysis such as HRV, a heuristic rule requires at least 10 complete cycles of the lowest observed frequency in the sampling period [22,34]. The 5-min recording of HRV can resolve frequencies down to 0.033Hz in light of this rule. Therefore, we included both LF% and LF/HF based on the LF spectrum ranging from 0.04–0.15 Hz. Whereas, we did not use VLF power with 0.003–0.04 Hz as the contribution from VLF is undersampled in this 5-min recording of HRV.

Increased HF, which represents vagal modulation, was found to occur during the two consecutive post-call days. Vagal activity has been related to sleepiness or sleep propensity [18,35], and a previous study found that parasympathetic activity predominates before people fall asleep [36]. Therefore, residents’ increased HF, which suggested sleepiness, would be an important indicator to monitor the risk of medical errors, which may jeopardize patient safety [37,38,39,40]. Residents’ increased HF during working hours shifted from interns’ increased HF during sleep in our earlier study [18]. These findings suggested a chronic sleep debt resulted from an extremely heavy workload in terms of hours worked throughout medical training. We attribute this increased HF to the heavy working hours rather than sleep deprivation during on-call duty. Although there was significantly shorter sleep during on-call night shifts, the difference is one hour less than the pre-call day or the post-call days. In addition, although a duty hour restriction lets residents leave work after noon on the post-call day, HF still increased during the second post-call day.

No HRV indices changed during the night shift (the 9th to the 16th consecutive working hours) or post-call (the 28th to the 33rd hours) rest, compared to the same time period on the other days. These results revealed that the post-call effects, either long working hours or post-call rest, would not reflect immediately on HRV. A recent study also found no significant differences in key HRV measures in obstetricians working 14 h versus 24 h in labor and delivery. These findings, as well as the aforementioned, significantly reduced LF and increased HF on the first and second post-call days, which suggested that researchers should record HRV over two post-call days.

Our study demonstrated that male residents had higher sympathetic modulation (LF/HF and LF%) than female residents did. This result is consistent with a previous epidemiology survey, in which men had a predominant sympathetic modulation compared with women [41]. The mixed model controlled for variations between subjects. Analysis of each subject as his/her own comparator strengthened the results. However, the short-term effects did not conflict with our previous findings that female interns’ HRV measurements did not significantly change through the 12-month internship, whereas male interns’ HF decreased at 9 and 12 months. Epidemiological studies have revealed that the incidence of cardiovascular disease is lower in premenopausal women than in age-matched men [41]. Moreover, cardiac vagal modulation, a protective index of the severity of CVD [42], is more predominant among premenopausal women than among men [25]. Future studies should focus on the progression and potential protective mechanisms from HRV changes, shifting from the present study’s short-term changes (within days) to long-term changes (as measured in months or even years). In addition, there is a need to investigate the role of hormones, such as estrogen, progesterone, and cortisol, in the stress response of the different genders, and the association between hormones and cardiac autonomic modulation. There are several methodological limitations that should be noted when interpreting our findings. Firstly, we could not control the quality of workload and sleep time during on-call duties in this naturalistic setting. These variations and the relatively small sample size could potentially diminish the significance of the HRV changes during the night shift and post-call rest. Secondly, our study lacks residents’ performance tests and cardiovascular biomarkers to link the HRV changes in this study. Neither did we account for psychological measurements, such as depression, anxiety, and burnout symptoms, which significantly increased during medical internship in our previous studies [17,18,43,44,45,46,47]. A recent meta-analysis demonstrated that physicians with a positive screening for depressive symptoms are at higher risk for medical errors [48]. Thirdly, although we demonstrate the impact of on-call duty on HRV, there was no control group to compare residents with and without the current working hours restrictions; however, in HRV research among medical trainees control groups have rarely been employed [49].

## 5. Conclusions

In conclusion, our studies suggest the current duty-hour guideline, which limits workweeks to 80 h and shifts to 28 h, resulted in reduced sympathetic modulation and increased parasympathetic modulation during the two days following on-call duty. Our findings provide insights into electrophysiological biomarkers to monitor physicians’ work overload and may facilitate the improvement of policies on duty-hour restrictions.

## Figures and Tables

**Table 1:** Regression coefficients resulting from the construction of a linear mixed-effect model 1 of the HRV among all participants. Four HRV measurements of outcome of interest are R-R interval, HF, LF%, and LF/HF. In this model, the major determinants—gender, duty day, and circadian period—were included as fixed effects. The pre-call day and the morning period were self-control references.

	RR (ms)			HF [ln (ms^2^)]			LF %			LF/HF [ln(ratio)]		
Coefficient	Estimate	SE	*p* Value	Estimate	SE	*p* Value	Estimate	SE	*p* Value	Estimate	SE	*p* Value
Intercept	703.02	26.48	<0.001	5.69	0.16	<0.001	50.56	2.67	<0.001	0.63	0.1	<0.001
Male vs. female	4.16	32.41	0.898	−0.25	0.19	0.185	16.63	2.79	<0.001	0.42	0.11	<0.001
On-call day vs. pre-call day	−8.61	9.74	0.377	0.04	0.08	0.607	−1.74	1.94	0.371	0.03	0.07	0.664
Post-call day vs. pre-call day	5.94	9.74	0.542	0.17	0.08	0.041	−6.39	1.94	0.001	−0.11	0.07	0.118
Second day post-call vs. pre-call day	−2.24	9.74	0.818	0.31	0.08	<0.001	−4.69	1.94	0.016	−0.16	0.07	0.023
Afternoon (12:00–16:59) vs. morning (08:00–11:59)	13.97	8.44	0.098	0.06	0.07	0.393	−2.74	1.68	0.104	−0.1	0.06	0.099
Night (17:00–23:59) vs. morning (08:00–11:59)	35.82	8.44	<0.001	0.11	0.07	0.135	−2.65	1.68	0.115	−0.08	0.06	0.203

**Table 2:** Regression coefficients resulting from the construction of a linear mixed-effect model 2 of the HRV among all participants. In this model, we additionally examined the effects of duty night and post-call rest.

	RR (ms)			HF [ln (ms^2^)]			LF %			LF/HF [ln(ratio)]		
Coefficient	Estimate	SE	*p* Value	Estimate	SE	*p* Value	Estimate	SE	*p* Value	Estimate	SE	*p* Value
Intercept	704.42	26.55	<0.001	5.7	0.16	<0.001	50.23	2.69	<0.001	0.62	0	<0.001
Male vs. female	4.16	32.41	0.898	−0.25	0.19	0.185	16.63	2.79	<0.001	0.42	0.11	<0.001
On-call day vs. pre-call day	−10.82	11.33	0.34	−0.03	0.1	0.761	−0.86	2.26	0.703	0.02	0.108	0.768
Post-call day vs. pre-call day	2.54	11.33	0.822	0.18	0.1	0.06	−5.95	2.26	0.009	−0.07	0.08	0.383
Second day post-call vs. pre-call day	−2.24	9.78	0.819	0.32	0.08	<0.001	−4.69	1.95	0.016	−0.16	0.07	0.023
Duty night	6.61	17.18	0.7	0.22	0.15	0.138	−2.63	3.43	0.442	0.09	0.12	0.876
Post-call rest	10.18	17.18	0.553	−0.03	0.15	0.814	−1.32	3.43	0.7	−0.12	0.12	0.349
Afternoon (12:00–16:59) vs. morning (08:00–11:59)	11.42	9.49	0.229	0.07	0.08	0.385	−2.41	1.89	0.203	−0.07	0.07	0.297
Night (17:00–23:59) vs. morning (08:00–11:59)	34.16	9.49	<0.001	0.05	0.08	0.506	−2	1.89	0.292	−0.08	0.07	0.229

## Abbreviations

ACGME	Accreditation Council for Graduate Medical Education
ANS	autonomic nervous system
CVD	cardiovascular disease
ECG	electrocardiographic
HF	high-frequency
HRV	Heart Rate Variability
LF	low-frequency
PGY	post-graduate year
TP	total power
TST	Total sleep time
VLF	very low frequency
VT	ventricular tachycardia

## References

[1] Arora V.M., Georgitis E., Siddique J., Vekhter B., Woodruff J.N., Humphrey H.J., Meltzer D.O. (2008). Association of workload of on-call medical interns with on-call sleep duration, shift duration, and participation in educational activities. JAMA.

[2] Kang M.Y., Park H., Seo J.C., Kim D., Lim Y.H., Lim S., Cho S.H., Hong Y.C. (2012). Long working hours and cardiovascular disease: A meta-analysis of epidemiologic studies. J. Occup Environ. Med..

[3] Vyas M.V., Garg A.X., Iansavichus A.V., Costella J., Donner A., Laugsand L.E., Janszky I., Mrkobrada M., Parraga G., Hackam D.G. (2012). Shift work and vascular events: Systematic review and meta-analysis. BMJ.

[4] Yang H., Durocher J.J., Larson R.A., Dellavalla J.P., Carter J.R. (2012). Total sleep deprivation alters cardiovascular reactivity to acute stressors in humans. J. Appl. Physiol. (1985).

[5] Sabanayagam C., Shankar A. (2010). Sleep duration and cardiovascular disease: Results from the National Health Interview Survey. Sleep.

[6] Shankar A., Koh W.P., Yuan J.M., Lee H.P., Yu M.C. (2008). Sleep duration and coronary heart disease mortality among Chinese adults in Singapore: A population-based cohort study. Am. J. Epidemiol..

[7] Heslop P., Smith G.D., Metcalfe C., Macleod J., Hart C. (2002). Sleep duration and mortality: The effect of short or long sleep duration on cardiovascular and all-cause mortality in working men and women. Sleep Med..

[8] Mansukhani M.P., Kolla B.P., Surani S., Varon J., Ramar K. (2012). Sleep deprivation in resident physicians, work hour limitations, and related outcomes: A systematic review of the literature. Postgrad. Med..

[9] Nasca T.J., Day S.H., Amis E.S., Force A.D.H.T. (2010). The new recommendations on duty hours from the ACGME Task Force. N. Engl. J. Med..

[10] Ministry of Health and Welfare Medical Residents’ Labor Rights Protection Guideline. https://www.health.taichung.gov.tw/media/302252/481916431154.pdf..

[11] Patel M.S., Volpp K.G., Small D.S., Hill A.S., Even-Shoshan O., Rosenbaum L., Ross R.N., Bellini L., Zhu J., Silber J.H. (2014). Association of the 2011 ACGME resident duty hour reforms with mortality and readmissions among hospitalized Medicare patients. JAMA.

[12] Bilimoria K.Y., Chung J.W., Hedges L.V., Dahlke A.R., Love R., Cohen M.E., Hoyt D.B., Yang A.D., Tarpley J.L., Mellinger J.D. (2016). National Cluster-Randomized Trial of Duty-Hour Flexibility in Surgical Training. N. Engl. J. Med..

[13] Rajaram R., Chung J.W., Jones A.T., Cohen M.E., Dahlke A.R., Ko C.Y., Tarpley J.L., Lewis F.R., Hoyt D.B., Bilimoria K.Y. (2014). Association of the 2011 ACGME resident duty hour reform with general surgery patient outcomes and with resident examination performance. JAMA.

[14] Volpp K.G., Rosen A.K., Rosenbaum P.R., Romano P.S., Even-Shoshan O., Canamucio A., Bellini L., Behringer T., Silber J.H. (2007). Mortality among patients in VA hospitals in the first 2 years following ACGME resident duty hour reform. JAMA.

[15] Volpp K.G., Rosen A.K., Rosenbaum P.R., Romano P.S., Even-Shoshan O., Wang Y., Bellini L., Behringer T., Silber J.H. (2007). Mortality among hospitalized Medicare beneficiaries in the first 2 years following ACGME resident duty hour reform. JAMA.

[16] Lin Y.H., Ho Y.C., Lin S.H., Yeh Y.H., Liu C.Y., Kuo T.B., Yang C.C., Yang A.C. (2013). On-call duty effects on sleep-state physiological stability in male medical interns. PLoS ONE.

[17] Lin Y.H., Chen C.Y., Lin S.H., Liu C.H., Weng W.H., Kuo T.B., Yang C.C. (2013). Gender differences in cardiac autonomic modulation during medical internship. Psychophysiology.

[18] Lin Y.H., Kuo T.B., Ho Y.C., Lin S.H., Liu C.Y., Yang C.C. (2012). Physiological and psychological impacts on male medical interns during on-call duty. Stress.

[19] Hirsch J.A., Bishop B. (1981). Respiratory sinus arrhythmia in humans: How breathing pattern modulates heart rate. Am. J. Physiol.

[20] Kuo T.B., Lai C.J., Huang Y.T., Yang C.C. (2005). Regression analysis between heart rate variability and baroreflex-related vagus nerve activity in rats. J. Cardiovasc. Electrophysiol..

[21] Malliani A., Pagani M., Lombardi F., Cerutti S. (1991). Cardiovascular neural regulation explored in the frequency domain. Circulation.

[22] Task Force of the European Society of Cardiology and the North American Society of Pacing and Electrophysiology (1996). Heart rate variability: Standards of measurement, physiological interpretation and clinical use. Task Force of the European Society of Cardiology and the North American Society of Pacing and Electrophysiology. Circulation.

[23] Kuo T.B., Yang C.C. (2009). Frequency domain analysis of electrooculogram and its correlation with cardiac sympathetic function. Exp. Neurol..

[24] Kuo T.B., Chan S.H. (1993). Continuous, on-line, real-time spectral analysis of systemic arterial pressure signals. Am. J. Physiol..

[25] Kuo T.B., Lin T., Yang C.C., Li C.L., Chen C.F., Chou P. (1999). Effect of aging on gender differences in neural control of heart rate. Am. J. Physiol..

[26] Quintana Daniel S., Heathers J.A. (2014). Considerations in the assessment of heart rate variability in biobehavioral research. Front. Psychol.

[27] Furlan R., Barbic F., Piazza S., Tinelli M., Seghizzi P., Malliani A. (2000). Modifications of cardiac autonomic profile associated with a shift schedule of work. Circulation.

[28] Selye H. (1950). The Physiology and Pathology of Exposure to Stress, a Treatise Based on the Concepts of the General-Adaptationsyndrome and the Diseases of Adaptation.

[29] Shusterman V., Aysin B., Gottipaty V., Weiss R., Brode S., Schwartzman D., Anderson K.P. (1998). Autonomic nervous system activity and the spontaneous initiation of ventricular tachycardia. ESVEM Investigators. Electrophysiologic Study Versus Electrocardiographic Monitoring Trial. J. Am. Coll. Cardiol..

[30] Lombardi F., Porta A., Marzegalli M., Favale S., Santini M., Vincenti A., De Rosa A. (2000). Implantable Cardioverter Defibrillator-Heart Rate Variability Italian Study, G., Heart rate variability patterns before ventricular tachycardia onset in patients with an implantable cardioverter defibrillator. Participating Investigators of ICD-HRV Italian Study Group. Am. J. Cardiol..

[31] Huikuri H.V., Valkama J.O., Airaksinen K.E., Seppanen T., Kessler K.M., Takkunen J.T., Myerburg R.J. (1993). Frequency domain measures of heart rate variability before the onset of nonsustained and sustained ventricular tachycardia in patients with coronary artery disease. Circulation.

[32] Heathers J.A.J. (2014). Everything Hertz: Methodological issues in short-term frequency-domain HRV. Front. Psychol..

[33] Kobayashi H., Park B.J., Miyazaki Y. (2012). Normative references of heart rate variability and salivary alpha-amylase in a healthy young male population. J. Physiol. Anthropol..

[34] Berntson G., Bigger J., Eckberg D., Grossman P., Kaufmann P., Malik M., Haikady N., Stephen W., Porges J., Philip S. (1997). Heart rate variability: Origins, methods, and interpretive caveats. Psychophysiol..

[35] Delamont R.S., Julu P.O., Jamal G.A. (1998). Sleep deprivation and its effect on an index of cardiac parasympathetic activity in early nonREM sleep in normal and epileptic subjects. Sleep.

[36] Kuo T.B., Shaw F.Z., Lai C.J., Yang C.C. (2008). Asymmetry in sympathetic and vagal activities during sleep-wake transitions. Sleep.

[37] Levine A.C., Adusumilli J., Landrigan C.P. (2010). Effects of reducing or eliminating resident work shifts over 16 h: A systematic review. Sleep.

[38] Moonesinghe S.R., Lowery J., Shahi N., Millen A., Beard J.D. (2011). Impact of reduction in working hours for doctors in training on postgraduate medical education and patients’ outcomes: Systematic review. BMJ.

[39] Qureshi A.U., Ali A.S., Hafeez A., Ahmad T.M. (2010). The effect of consecutive extended duty hours on the cognitive and behavioural performance of paediatric medicine residents. J. Pak. Med. Assoc..

[40] Ehara A. (2008). Are long physician working hours harmful to patient safety?. Pediatr. Int..

[41] Lerner D.J., Kannel W.B. (1986). Patterns of coronary heart disease morbidity and mortality in the sexes: A 26-year follow-up of the Framingham population. Am. Heart J..

[42] Singh J.P., Larson M.G., Tsuji H., Evans J.C., O’Donnell C.J., Levy D. (1998). Reduced heart rate variability and new-onset hypertension: Insights into pathogenesis of hypertension: The Framingham Heart Study. Hypertension.

[43] Lin Y.H., Lin S.H., Li P., Huang W.L., Chen C.Y. (2013). Prevalent hallucinations during medical internships: Phantom vibration and ringing syndromes. PLoS ONE.

[44] Lin Y.H., Chen C.Y., Li P., Lin S.H. (2013). A dimensional approach to the phantom vibration and ringing syndrome during medical internship. J. Psychiatry. Res..

[45] Chen C.Y., Lin S.H., Li P., Huang W.L., Lin Y.H. (2015). The role of the harm avoidance personality in depression and anxiety during the medical internship. Medicine..

[46] Liu C.H., Tang W.R., Weng W.H., Lin Y.H., Chen C.Y. (2016). The process of coping with stress by Taiwanese medical interns: A qualitative study. BMC Medical Res..

[47] Lin Y.H., Chen H.Y., Tsai S.L., Chang L.R., Chen P.C. (2019). A prospective study of the factors associated with life quality during medical internship. PLoS ONE.

[48] Pereira-Lima K., Mata D.A., Loureiro S.R., Crippa J.A., Bolsoni L.M., Sen S. (2019). Association Between Physician Depressive Symptoms and Medical Errors: A Systematic Review and Meta-analysis. JAMA Netw. Open.

[49] Thielmann B., Boeckelmann I. (2016). Heart Rate Variability as an Indicator of Mental Stress in Surgeons—A Review of the Literature. Zentralbl. Chir..

